# Detection of the Antiviral Drug Oseltamivir in Aquatic Environments

**DOI:** 10.1371/journal.pone.0006064

**Published:** 2009-06-26

**Authors:** Hanna Söderström, Josef D. Järhult, Björn Olsen, Richard H. Lindberg, Hiroaki Tanaka, Jerker Fick

**Affiliations:** 1 Department of Chemistry, Umeå University, Umeå, Sweden; 2 Section of Infectious Diseases, Department of Clinical Sciences, Uppsala University Hospital, Uppsala, Sweden; 3 Section for Zoonotic Ecology and Epidemiology, Kalmar University, Kalmar, Sweden; 4 Research Center of Environmental Quality Management, Kyoto University, Otsu, Japan; Naval Research Laboratory, United States of America

## Abstract

Oseltamivir (Tamiflu®) is the most important antiviral drug available and a cornerstone in the defence against a future influenza pandemic. Recent publications have shown that the active metabolite, oseltamivir carboxylate (OC), is not degraded in sewage treatment plants and is also persistent in aquatic environments. This implies that OC will be present in aquatic environments in areas where oseltamivir is prescribed to patients for therapeutic use. The country where oseltamivir is used most is Japan, where it is used to treat seasonal flu. We measured the levels of OC in water samples from the Yodo River system in the Kyoto and Osaka prefectures, Japan, taken before and during the flu-season 2007/8. No OC was detected before the flu-season but 2–58 ng L^−1^ was detected in the samples taken during the flu season. This study shows, for the first time, that low levels of oseltamivir can be found in the aquatic environment. Therefore the natural reservoir of influenza virus, dabbling ducks, is exposed to oseltamivir, which could promote the evolution of viral resistance.

## Introduction

Oseltamivir (Tamiflu®) is the most important of the few antiviral drugs available for treatment of seasonal flu and a cornerstone in the defense against a future influenza pandemic. Most governments have built their preparedness plans around stockpiling oseltamivir and it is recommended by WHO both as treatment and prophylaxis in a pandemic situation [1].

Influenza A virus is a zoonosis, with its natural reservoir in dabbling ducks [2]. It belongs to the *orthomyxoviridae*, a negative strand RNA virus family, which also includes influenza B and C; however the two latter are of less importance as human pathogens. The different influenza A virus subtypes are named after the type of two cell-surface glycoproteins, hemagglutinin (HA) and neuraminidase (NA) [3]. At present, there are two subtypes, H1N1 and H3N2, which cause the annual seasonal influenza epidemics [3]. All influenza infections render humoral immunologic memory, but antigenic changes are so frequent that previous infections often give only limited immunity to concurrent virus. This may be a viral strategy, where the low specificity of the virus polymerase generates frequent mismatches and a high rate of mutations. The influenza virus has another, more drastic way of genetic change, where genetic elements from two viruses infecting the same cell can be recombined. This process, termed ‘genetic reassortment’, promotes rapid evolutionary changes and is the key to the genesis of new strains of human influenza capable of causing a pandemic [3]–[5]. Both the ‘Asian flu’ in 1957 (H2N2) and the ‘Hong Kong flu’ in 1968 (H3N2) were reassortments between human-adapted seasonal influenza strains and contemporary avian strains [6].

Oseltamivir is a neuraminidase inhibitor administered orally as a prodrug, oseltamivir phosphate, which is converted to the active metabolite OC in the liver and then excreted without further metabolism through the urine [7]. In a previous study, we have shown that the active metabolite of oseltamivir, oseltamivir carboxylate (OC) is neither degraded nor removed in sewage treatment plants (STPs) [8]. Thus, we assumed that OC can be present in the aquatic environment. Recent publications show that OC is quite persistent in aquatic environments and is only removed by microbial degradation associated with sediment [9], [10]. A hypothesis has been presented that OC residues in the environment, either after usage during a pandemic or for treatment of seasonal influenza, could expose the natural reservoir of influenza virus, dabbling ducks, to low levels of this antiviral which could promote resistance development [8], [11]. OC usage during a pandemic could also have other ecotoxicological effects [12].

Japan is the top per-capita-consumer of oseltamivir; the manufacturer Roche estimates that 6 of 16 million infected Japanese were prescribed the drug during the 2004/05 flu season [13]. A study was conducted with the assumption that a) OC is detectable in Japanese waterways, b) levels increase at the peak of the flu season and c) levels are higher closer to the outlet of a STP. We chose to study the Yodo River system in the Kyoto and Osaka prefectures, a densely populated area located off-sea.

## Materials and Methods

### Sampling

**Figure 1:**
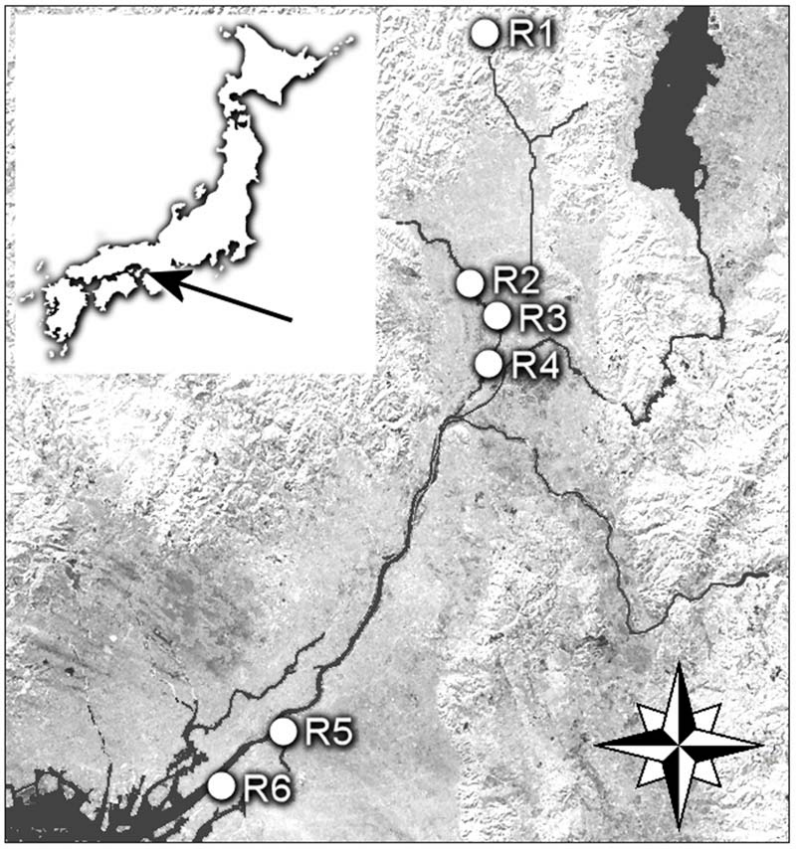
Sampling locations in Kyoto (R1–R4) and Osaka (R5–R6) prefectures, Japan.

Surface water was collected before (June 2007) and during (December 2007 and February 2008) the flu season 2007/8 from totally six sites (R1–R6) in three rivers of the Yodo River system (Fig. 1). R1 (N17° 32.396 E78° 14.590, Kyoto), R2 (N17° 34.422 E78° 21.359, Kyoto), R3 (N17° 34.422 E78° 21.359, Kyoto), R4 (N17° 34.451 E78° 21.390, Kyoto), R5 (N17° 33.165 E78° 19.990, Osaka) and R6 (N17° 36.845 E78° 11.598, Osaka). Repeated sampling was performed at sites R1, R2 and R4. R1 was located in a rural area of Kamo River, before the river enters more populated areas. In Katsura River samples were taken 1 km upstream (R2) and 2.5 km downstream (R4) of the outlet of one of the major STPs in Kyoto prefecture. In February 2008, additional sampling was performed closer to the STP (R3), 0.5 km downstream of its outlet, and in the Yodo River (R5 and R6) in the Osaka prefecture, respectively, where the water has run through almost all of the river system, collecting the sewage outlets from approximately 5 million inhabitants. Duplicate 500 ml samples were taken at each location; these were immediately frozen and kept at −18°C until analysis.

### Chemicals

Oseltamivir carboxylate (OC), (RO0640802-002; lot: 01007B243804) and Oseltamivir carboxylate labelled with deuterium (OC_D3_), (RO0604802-004; lot: 511-001-2197/4) were obtained from Roche (F. Hoffmann-La Roche Ltd, Basel, Switzerland). Formic acid, ammonium hydroxide 25% and methanol (HPLC-grade) were purchased from JT Baker (Deventer, the Netherlands), acetonitrile (HPLC-grade) from Fischer Chemicals (Zurich, Switzerland) and sulphuric acid from Merck (Darmstadt, Germany). The purified water (resistivity, 18.2 MΩ cm) was prepared by an ELGA MAXIMA HPLC ultra pure water system (ELGA, High Wycombe Bucks, UK). Standard stock solutions of OC and OC_D3_, 100 ng mL^−1^, were prepared in water (10 mL) and kept dark at 4°C.

### Sample pretreatment

Samples were filtered through 0.45 µm MF™-membrane filters (Millipore, Sundbyberg, Sweden) before acidification to pH 3 using sulphuric acid. The internal standard OC_D3_ was added to each sample to an amount of 500 ng. The Strata-X-C (200 mg, 6 mL) mixed mode cation exchange sorbent (Phenomenex, email: international@phenomenex.com) used for the solid phase extraction (SPE) was conditioned and equilibrated by 2.0 ml of methanol and 2.0 ml of deionized water. 500 mL of the samples were applied to the SPE columns at a flow rate of 5 mL min^−1^. Impurities were removed by 2.0 ml 0.1% sulphuric acid and the sorbents were dried (1 min at 10” Hg). Neutral and acidic components were removed by 2 mL of methanol and wasted, followed by elution of the analytes by 2 mL of 5% NH_4_OH in methanol. The eluates were evaporated to approximately 20 µl using air and then reconstituted in acetonitrile in water (1∶1), containing 0.1% formic acid, to a final extract volume of 1.0 ml.

### Liquid Chromatography-Mass Spectrometry

Sample extracts and calibration solutions (7.5 µl) were injected into a Waters Acquity™ Ultra Performance LC system connected to a Micromass Quattro Ultima triple quadruple mass spectrometer. OC and the internal standard, OC_D3_, were separated by using: equal amounts of the mobile phases H_2_0 and ACN, both containing 0.1% (v/v) formic acid; a flow rate of 0.200 ml min^−1^ and a Waters Acquity™ UPLC BEH C18 (2.1×50 mm, 1.7 µm particle size) analytical column. The electrospray was held in positive ion mode and the capillary and cone voltage were kept at 3.9 kV and 45 V, respectively. The source temperature was 110°C and the desolvation temperature was 310°C. The following transitions were monitored 284.9>196.6 (OC) and 287.9>199.9 (OC_D3_). Quantification was based on internal standard calibration and the peak area ratios of OC_D3_ and OC [8]. The linearity of the seven point calibration curve was acceptable (R^2^ above 0.997). LOQ was 1 ng L^−1^, determined by using the second point in the calibration curve.

## Results

**Figure 2:**
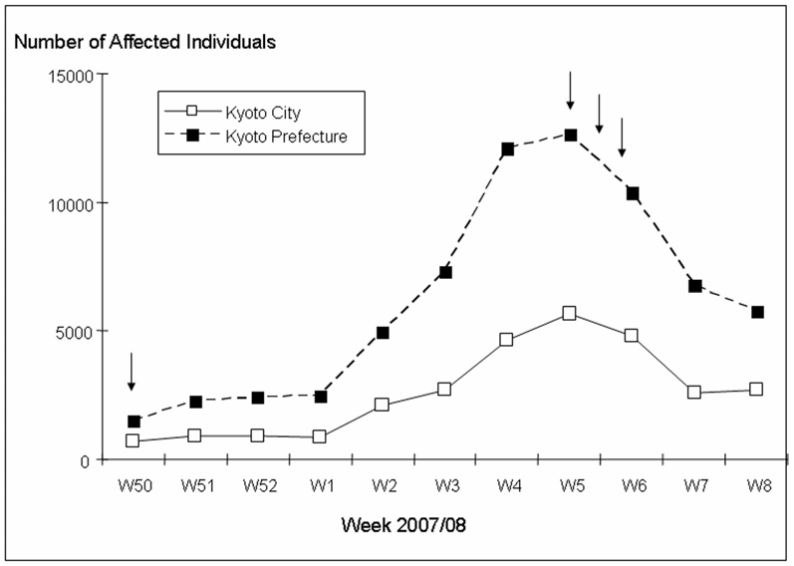
Number of individuals in Kyoto city and Kyoto prefecture affected by the flu during the flu season 07/08 [14]. Arrows indicate sampling events.

No OC was detected in the surface water collected in June 2007, during the influenza “off-season” (Table 1) and no influenza patients were reported this week [14]. In December, at the onset of the flu season (Fig. 2), 2 and 7 ng L^−1^ of OC was detected upstream (R2) and downstream (R4), respectively, of the STP (Table 1). At the peak of the flu-season, the levels of OC downstream the STP (R4) increased to 11 and 10 ng L^−1^, and OC levels of 19 ng L^−1^ was measured at the additional sampling point (R3) closer to the STP, while the levels of OC detected up-stream of the STP and in Kamo River were similar to those found in December (Table 1). At the peak of the flu-season, the OC concentrations in the Yodo River were also measured and the detected levels were 12 ng L^−1^ (R5) and 58 ng L^−1^ (R6) (Table 1). In Kyoto City, 6748 individuals were reported to have been affected by the flu during week 7 (11–17th of February) [14] (Fig. 2), i.e. the week we sampled water at site R2–R6. Assuming that approximately 40% received oseltamivir treatment [13], this corresponds to a weekly usage of 1.3 kg OC or 0.18 kg day^−1^ (estimated from 30% pediatric dosage, 70% adult dosage and 75% pro-drug conversion to OC, respectively). Calculations from the daily use and the average flow in Katsura River during the 12th of February (2291000 m^3^ day^−1^) yield a predicted environmental concentration (PEC) of 78 ng L^−1^. This PEC value is similar to the OC levels that we measured in Katsura River downstream of the STP on the same day. Furthermore, some of the levels detected in this study are close the IC_50_ (concentration that causes 50% inhibition) of OC, which however depends heavily on type of virus and exposure system, but such low levels as 80–230 ng L^−1^ have been reported [15], [16].

**Table 1:** Average water concentrations (ng L^−1^) of oseltamivir carboxylate (OC) measured in Kyoto (R1–R4) and Osaka (R5–R6) prefectures, Japan.

Date	Week	Kamo River	Katsura River			Yodo River	
		R1	R2	R3	R4	R5	R6
2007-06-13		nd	nd	-	nd	-	-
2007-12-17	50	-	2	-	7	-	-
2008-02-04	5	2	4	-	11	-	-
2008-02-12	6	-	4	19	10		
2008-02-15	6	-	-	-	-	12	58

## Discussion

We found that the active metabolite of oseltamivir is present in Japanese waterways at clearly detectable levels. The levels increase at the peak of the flu season and are in the same magnitude as the PEC value estimated from the total use of oseltamivir, and close to the IC_50_ of OC. Our results suggest that OC levels are higher closer to major STPs and further downstream in a river system. Subsequently, the natural reservoir of influenza virus, dabbling ducks [2], living in the aquatic environments in Japan or another area where oseltamivir is used widely, are exposed to OC. As influenza in dabbling ducks is a gastrointestinal infection, their bowel can contain replicating virus as well as oseltamivir, which could promote the evolution of viral resistance.

Strain-specific vaccines will not be available at the start of a pandemic. The only intervention possible in the absence of vaccines would be the use of antivirals. Earlier pandemic influenza viruses have contained genetic material from avian strains and if oseltamivir-resistance is induced and transferred from the avian reservoir to a virus with pandemic potential there is an imminent risk that one of the cornerstones in pandemic preparedness have been disarmed. It is therefore essential to investigate the biological significance of the observed levels of OC in the environment.
